# HILPDA Is a Prognostic Biomarker and Correlates With Macrophage Infiltration in Pan-Cancer

**DOI:** 10.3389/fonc.2021.597860

**Published:** 2021-03-18

**Authors:** Chengdong Liu, Xiaohan Zhou, Hanyi Zeng, Dehua Wu, Li Liu

**Affiliations:** ^1^Department of Infectious Diseases, Nanfang Hospital, Southern Medical University, Guangzhou, China; ^2^Department of Radiation Oncology, Nanfang Hospital, Southern Medical University, Guangzhou, China

**Keywords:** HILPDA, pan-cancer, tumor associated macrophages, TCGA, immunossuppression

## Abstract

**Background:** The protein hypoxia-inducible lipid droplet-associated (HILPDA) is differentially expressed in various tumors. However, its role and correlation with immune cell infiltration in most tumors remain unclear.

**Methods:** HILPDA expression was analyzed in pan-cancer data from The Cancer Genome Atlas (TCGA) database. The influence of HILPDA in clinical prognosis was evaluated using clinical survival data from TCGA. Enrichment analysis of HILPDA was conducted using the R package “clusterProfiler.” We downloaded the immune cell infiltration score of TCGA samples from published articles and analyzed the correlation between the magnitude of immune cell infiltration and HILPDA expression.

**Results:** HILPDA was highly expressed and associated with worse overall survival, disease-specific survival, and progression-free interval in most tumor types. In addition, HILPDA expression was significantly associated with the glycolysis pathway and infiltration of immune cells. Tumor-associated macrophage (TAM) infiltration increased in tissues with high HILPDA expression in most tumor types. Immunosuppressive genes, such as *PD-L1, PD-1, TGFB1*, and *TGFBR1* were positively correlated with HILPDA.

**Conclusions:** Our study suggests that HILPDA is a marker of poor prognosis. High HILPDA may contribute to TAM infiltration and be associated with tumor immunosuppression status.

## Introduction

Hypoxia-inducible lipid droplet associated protein (HILPDA) plays an oncogenic role in various tumor types. For example, HILPDA is overexpressed in colorectal cancer and promotes cancer progression via hypoxia-dependent and independent pathways (1). Interestingly, high HILPDA expression was reported to predict worse patient survival in renal cell carcinoma, in which it may become a potential target for molecular therapy (2). However, the roles of HILPDA in most tumor types remain unclear.

Complex tumor microenvironment (TME), especially tumor immune microenvironment (TIME), is the main factor for poor prognosis of tumor patients (3). Tumor associated macrophages (TAMs) constitute the plasticity and heterogeneity of TME, which can account for 50% of some solid tumors (4). TAMs, especially M2-like TAMs, play an important role in tumor progression. Many oncogenes can promote the infiltration of TAMs in TME to accelerate tumor progression. However, the association between HILPDA expression and the infiltration of TAMs has not been explored.

In this study, we evaluated HILPDA expression in different tumor types from The Cancer Genome Atlas (TCGA) database and its association with tumor stage and prognosis of patients. We found that HILPDA is overexpressed in 14 tumor types. Additionally, high HILPDA expression was associated with worse overall survival (OS), disease-specific survival (DSS), and progression-free intervals (PFI) in most tumor types. HILPDA was predicted to participate in pathways related to the cell cycle and tumor immunity. As of immune cell infiltration is an important prognostic factor in tumor progression (5, 6), we examined the correlation between HILPDA expression and immune cell infiltration score and found that tumor associated macrophages (TAM) infiltration significantly increased in tissues with high HILPDA expression. Moreover, HILPDA was positively correlated with immunosuppressive gene, such as PD-L1, PD-1, TGFB1, and TGFBR1. Our results offer novel insights into the functional role of HILPDA and further highlight a potential mechanistic basis whereby HILPDA influences TAM infiltration and immunosuppressive gene expression in tumor microenvironment.

## Materials and Methods

### Data Collection and Analysis of HILPDA Expression

HILPDA expression profiles and clinical information of TCGA pan-cancer data were downloaded from the UCSC Xena (https://xenabrowser.net/datapages/) database. A total of 10496 patients with expression profiles and corresponding clinical data were included in our study. For HILPDA expression analysis in paired tumor and normal tissues, we selected a total of 1,362 patients with expression profiles of both tumor and adjacent normal tissues. For HILPDA expression analysis in different WHO stages, we selected a total of 7,105 patients with completed stage information. For survival analysis, 9,637, 9,165, and 9,479 patients with overall survival, disease-specific survival, and progression-free interval information were selected, respectively.

### Correlation and Enrichment Analyses

The correlation analysis of HILPDA was performed using TCGA LIHC data. The Pearson correlation coefficient was calculated. The top 300 genes most positively correlated with HILPDA were selected for enrichment analysis to reflect the function of HILPDA. Gene Set Variation Analysis (GSVA) was conducted using the R package “GSVA” to calculate the pathway score of each sample based on the MSigDB database v7.1 (https://www.gsea-msigdb.org/gsea/msigdb/index.jsp). Gene set enrichment analyses (GSEA) were conducted using the R package “clusterProfiler,” with the following parameters: nPerm = 1,000, minGSSize = 10, maxGSSize = 1,000, and *p*-value-Cutoff = 0.05.

### Immune Cell Infiltration

TCGA pan-cancer immune cell infiltration score was downloaded from the published study “The Immune Landscape of Cancer” (7), in which immune cell infiltration score was estimated using CIBERSORT. Samples of TCGA were divided into two groups (high HILPDA group and low HILPDA group) based on the median of HILPDA expression to compare the level of immune cell infiltration.

### Tumor Mutation Burden Calculation

TCGA somatic mutation data was downloaded from the UCSC XENA database. Tumor mutation burden (TMB) was calculated as the number of mutated bases per million bases, based on somatic mutation data in each tumor. The TMB results are shown in Supplementary Table 1.

### Statistical Analysis

Data are presented as the mean ± standard deviation (SD). Student's *t*-test (two-tailed) was used to analyze differences between two groups using R software (version: 3.6.2). *p* < 0.05 was considered statistically significant: ^*^p < 0.05; ^**^p < 0.01; ^***^p < 0.001; and ^****^p < 0.0001.

### Human Tissue Samples

The experiments involving human samples in our study were in accordance with the principles of the Declaration of Helsinki, and approved by the Institutional Review Board of Nanfang Hospital, Southern Medical University of Guangdong, China (NFEC-201208-K3). A total of 13 liver cancer and paired non-cancerous tissues were collected and used for qRT-PCR.

### qRT-PCR

TRIzol® reagent (TaKaRa, Tokyo, Japan), PrimeScript™ 1st Strand cDNA Synthesis Kit (TaKaRa, Tokyo, Japan), and SYBR® Green PCR kit (TaKaRa, Tokyo, Japan) were used to perform the extraction of total RNA, synthesization of First-strand cDNA, and Real-time PCR, respectively. Primers as follows:

ACTB:

Forward Primer CATGTACGTTGCTATCCAGGC,

Reverse Primer CTCCTTAATGTCACGCACGAT

HILPDA:

Forward Primer AAGCATGTGTTGAACCTCTACC

Reverse Primer TGTGTTGGCTAGTTGGCTTCT

CD274:

Forward Primer TGGCATTTGCTGAACGCATTT

Reverse Primer TGCAGCCAGGTCTAATTGTTTT

TGFB1:

Reverse Primer TGCAGCCAGGTCTAATTGTTTT

Reverse Primer GTGGGTTTCCACCATTAGCAC.

## Results

### HILPDA Expression Is High in Several Tumor Types and Correlates With Clinical Stage

**Figure 1:**
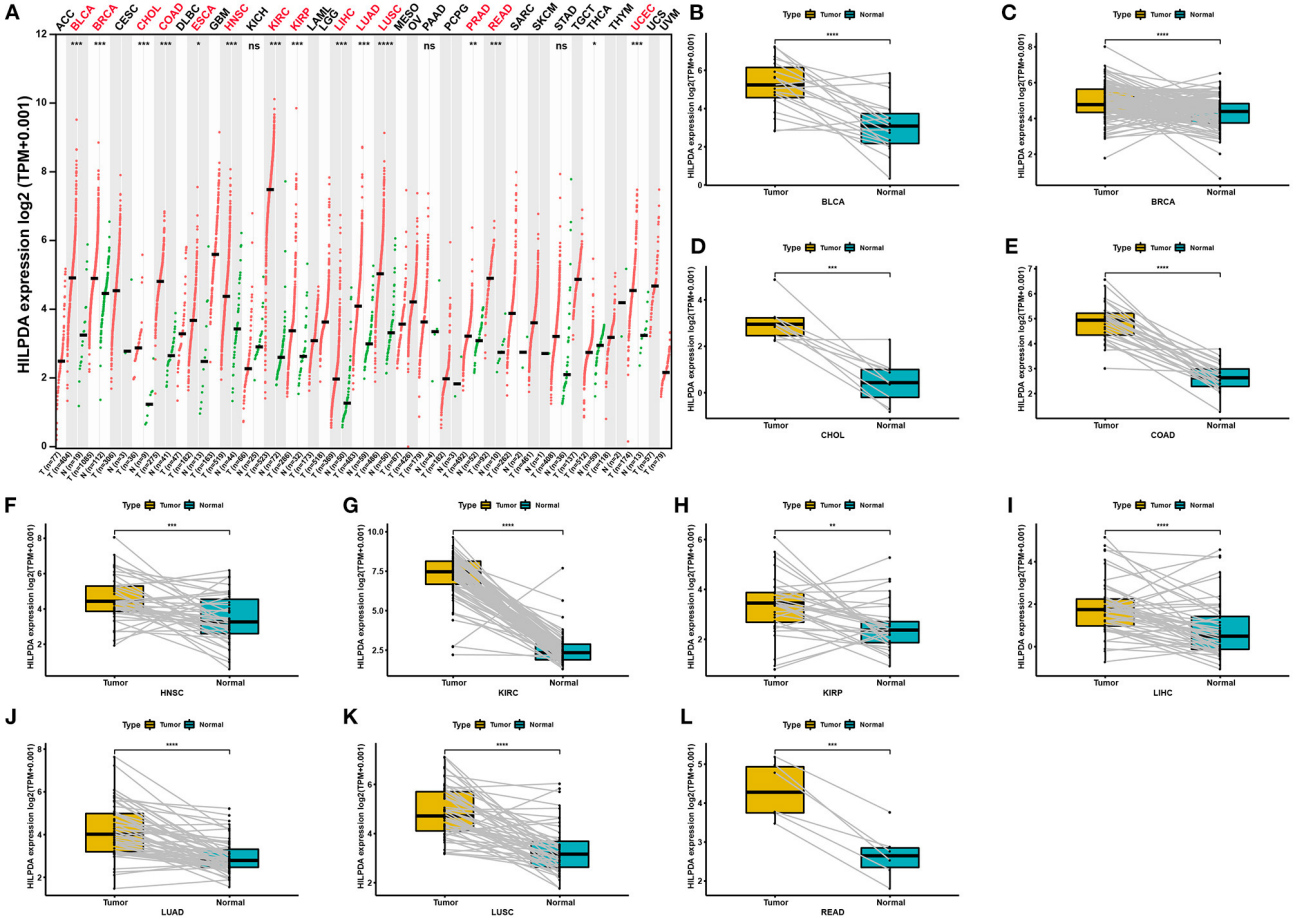
Pan-cancer HILPDA expression analysis. **(A)** HILPDA expression in tumor and normal tissues in pan-cancer data of TCGA using GEPIA2 database. **(B–L)** HILPDA expression in indicated paired tumor and normal tissues in pan-cancer data of TCGA. Gray lines connect paired tissues. Data shown as mean ± SD. *p < 0.05, **p < 0.01, ***p < 0.001, ****p < 0.0001.

**Figure 2:**
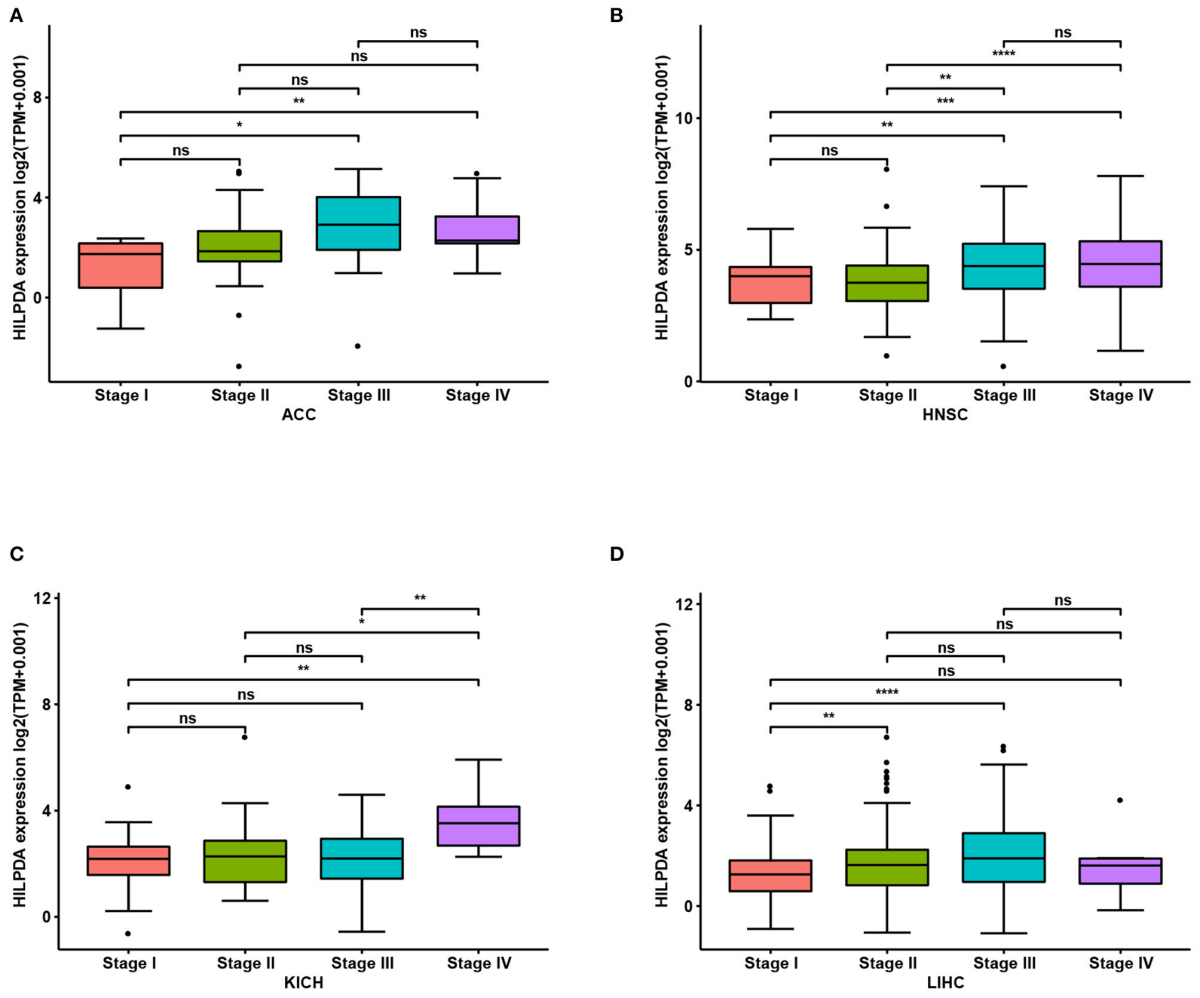
Association between HILPDA expression and tumor stages. **(A–D)** HILPDA expression in different stages in indicated tumor types. Data shown as mean ± SD. *p < 0.05, **p < 0.01, ***p < 0.001, ****p < 0.0001.

We first assessed HILPDA expression in pan-cancer data from TCGA. The analysis results revealed that HILPDA expression was higher in 14 tumors, including Bladder Urothelial Carcinoma (BLCA), Breast Invasive Carcinoma (BRCA), Cholangiocarcinoma (CHOL), Colon Adenocarcinoma (COAD), Esophageal Carcinoma (ESCA), Head and Neck Squamous Cell Carcinoma (HNSC), Kidney Renal Clear Cell Carcinoma (KIRC), Kidney Renal Clear Cell Carcinoma (KIRP), Liver Hepatocellular Carcinoma (LIHC), Lung Adenocarcinoma (LUAD), Lung Squamous Cell Carcinoma (LUSC), Prostate Adenocarcinoma (PRAD), Prostate Adenocarcinoma (READ), and Uterine Corpus Endometrial Carcinoma (UCEC), while lower expression was observed in Thyroid Carcinoma (THCA) (Figure 1A). For paired tumors and normal tissues, HILPDA was overexpressed in tumor tissues of BLCA, BRCA, CHOL, COAD, HNSC, KIRC, KIRP, LIHC, LUAD, LUSC, and READ (Figures 1B–L). In addition, the expression of HILPDA was closely related to the clinical stage, being higher in patients with relatively high stages of several tumor types, including Adrenocortical Carcinoma (ACC), HNSC, Kidney Chromophobe (KICH), and LIHC (Figures 2A–D).

### HILPDA High Expression Correlates With Poor Cancer Prognosis

**Figure 3:**
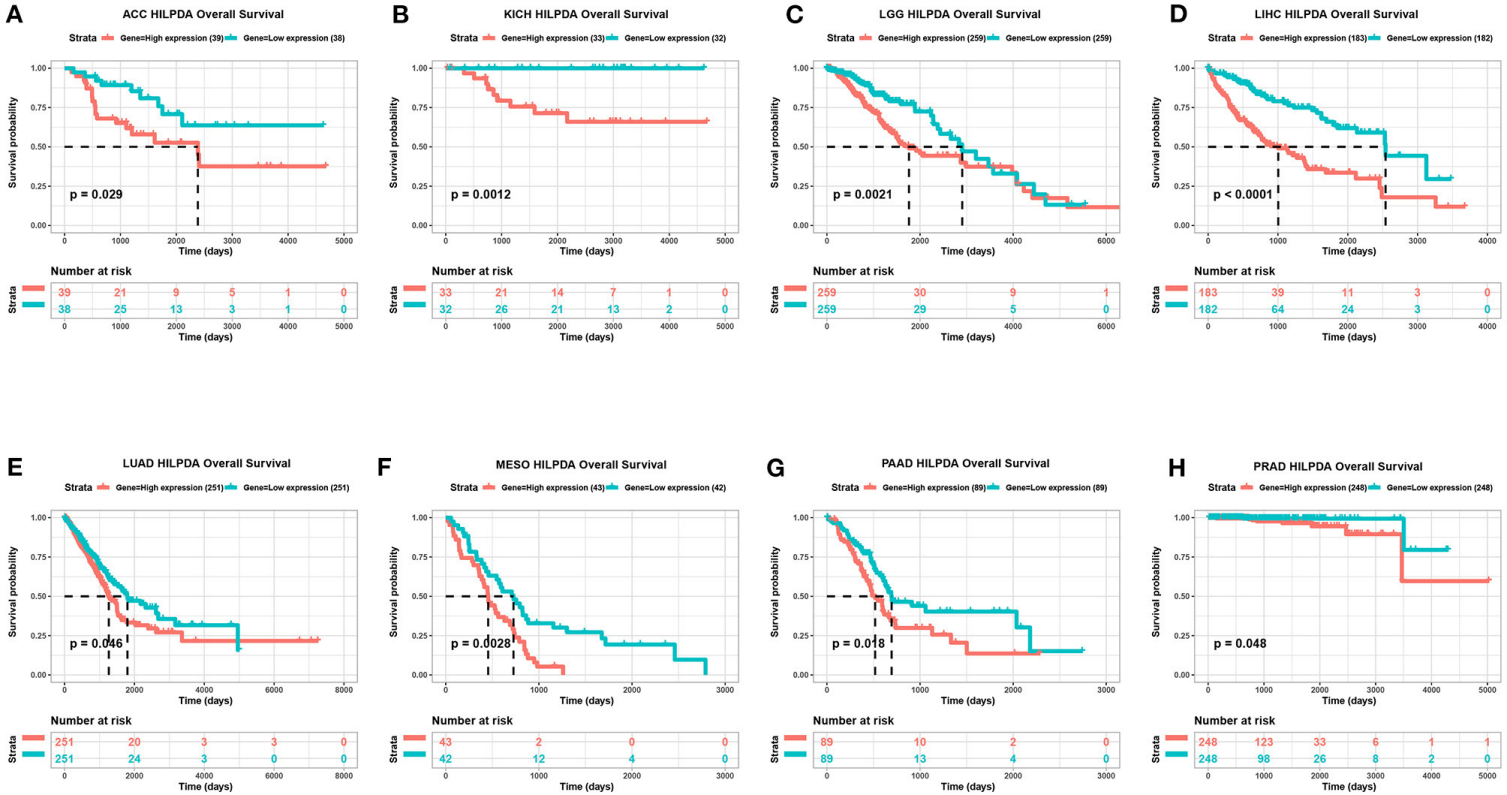
Association between HILPDA expression and OS of cancer patients. **(A–H)** Kaplan–Meier analysis of overall survival in 33 TCGA tumor types, group division was based on the median of HILPDA expression. Meaningless results are not shown.

**Figure 4:**
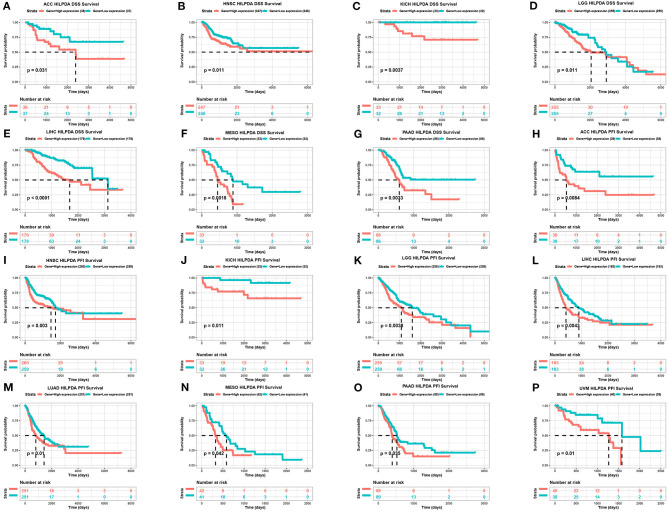
Association between HILPDA expression and DSS and PFI of cancer patients. **(A–G)** Kaplan–Meier analysis of DSS and **(H–P)** PFI in 33 TCGA tumor types, group division was based on the median of HILPDA expression. Meaningless results are not shown.

To evaluate the value of HILPDA in predicting the prognosis of cancer patients, the association between its expression and OS, DSS, and PFI was analyzed in TCGA cohort. Higher expression of HILPDA was significantly associated with worse OS in ACC (*p* = 0.029), KICH (*p* = 0.0012), LGG (*p* = 0.00097), LIHC (*p* < 0.0001), LUAD (*p* = 0.046), MESO (*p* = 0.0028), PAAD (*p* = 0.018), and PRAD (*p* < 0.048) (Figures 3A–H). Similarly, higher HILPDA expression was significantly associated with a reduction in DSS in ACC (*p* = 0.031), HNSC (*p* = 0.011), KICH (*p* = 0.0037), LGG (*p* = 0.011), LIHC (*p* < 0.0001), MESO (*p* < 0.0018), and PAAD (*p* = 0.0033) (Figures 4A–G). In addition, the PFI was reduced in the high HILPDA expression groups in ACC (*p* = 0.0084), HNSC (*p* = 0.003), KICH (*p* = 0.011), KIRC (*p* = 0.005), LGG (*p* = 0.0038), LIHC (*p* = 0.0043), LUAD (*p* = 0.01), MESO (*p* = 0.042), PAAD (*p* = 0.035), and UVM (*p* = 0.01) (Figures 4H–P).

### HILPDA Is Involved in Pathways Related to Malignancy

**Figure 5:**
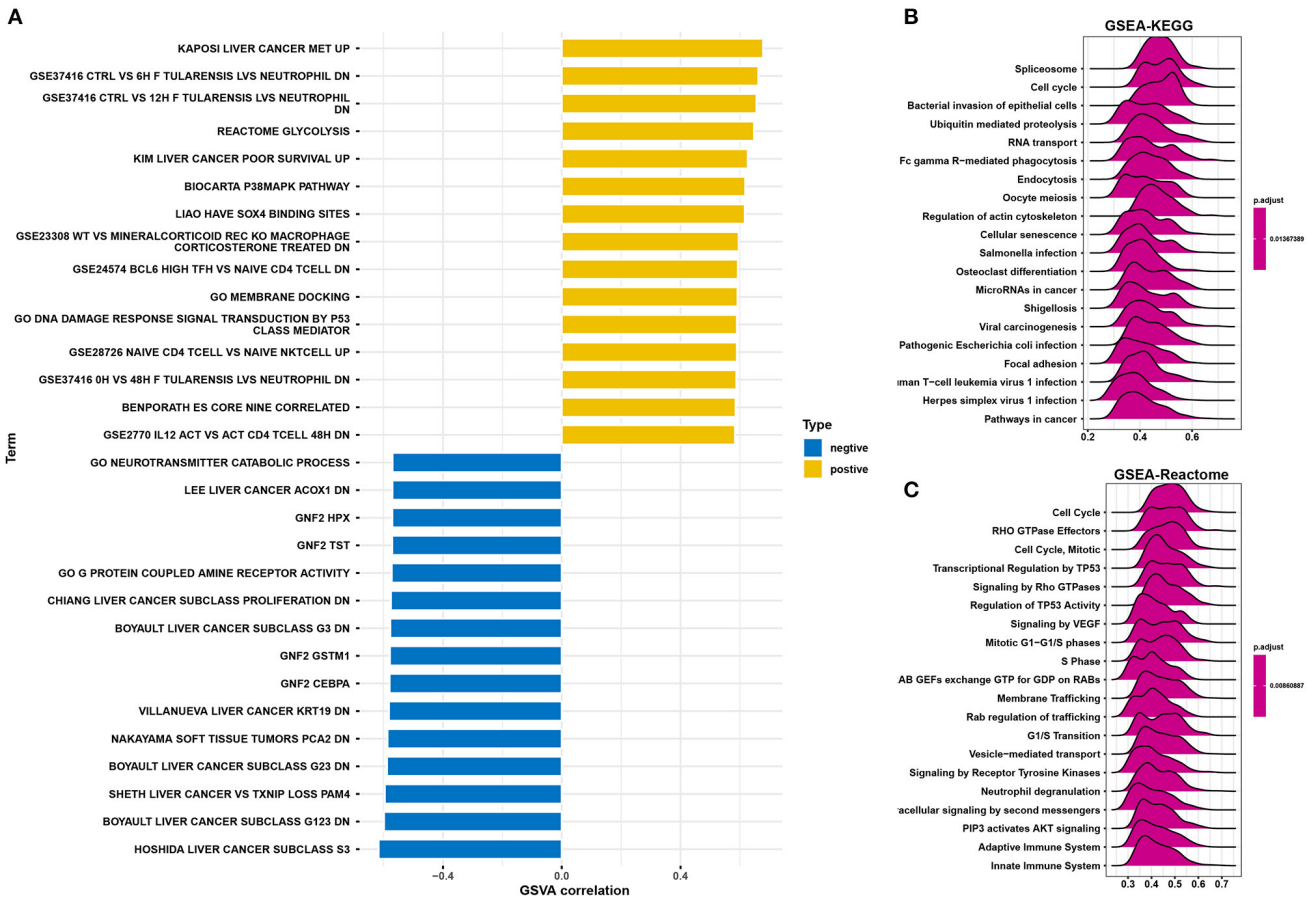
Function and pathway enrichment analysis of HILPDA. **(A)** Correlation analysis results of GSVA and HILPDA expression. **(B)** KEGG results of HILPDA GSEA in TCGA LIHC cohort. **(C)** Reactome results of HILPDA GSEA in TCGA LIHC cohort.

To predict the functions of HILPDA, we conducted a GSVA analysis based on gene sets from the MSigDB database v7.1. Results showed the scores of “Liver cancer MET up,” “Glycolysis,” and “Liver cancer poor survival up” pathways were positively correlated with HILPDA expression, indicating a role in these malignant processes (Figure 5A). We further conducted a GSEA analysis of HILPDA using TCGA LIHC data. The GSEA results showed that cell cycle-related pathways, including the cell cycle pathway in Kyoto Encyclopedia of Genes and Genomes (KEGG) and cell cycle, transcriptional regulation by P53, mitotic G-G1/S phases, and S phase in Reactome, were significantly enriched (Figures 5B,C). These results suggest that HILPDA is associated with many malignancy-related pathways, especially those related to the cell cycle and glycolysis.

### High HILPDA Expression Correlates With High Immune Cell Infiltration and Immunosuppressive Genes in Most Tumors

**Figure 6:**
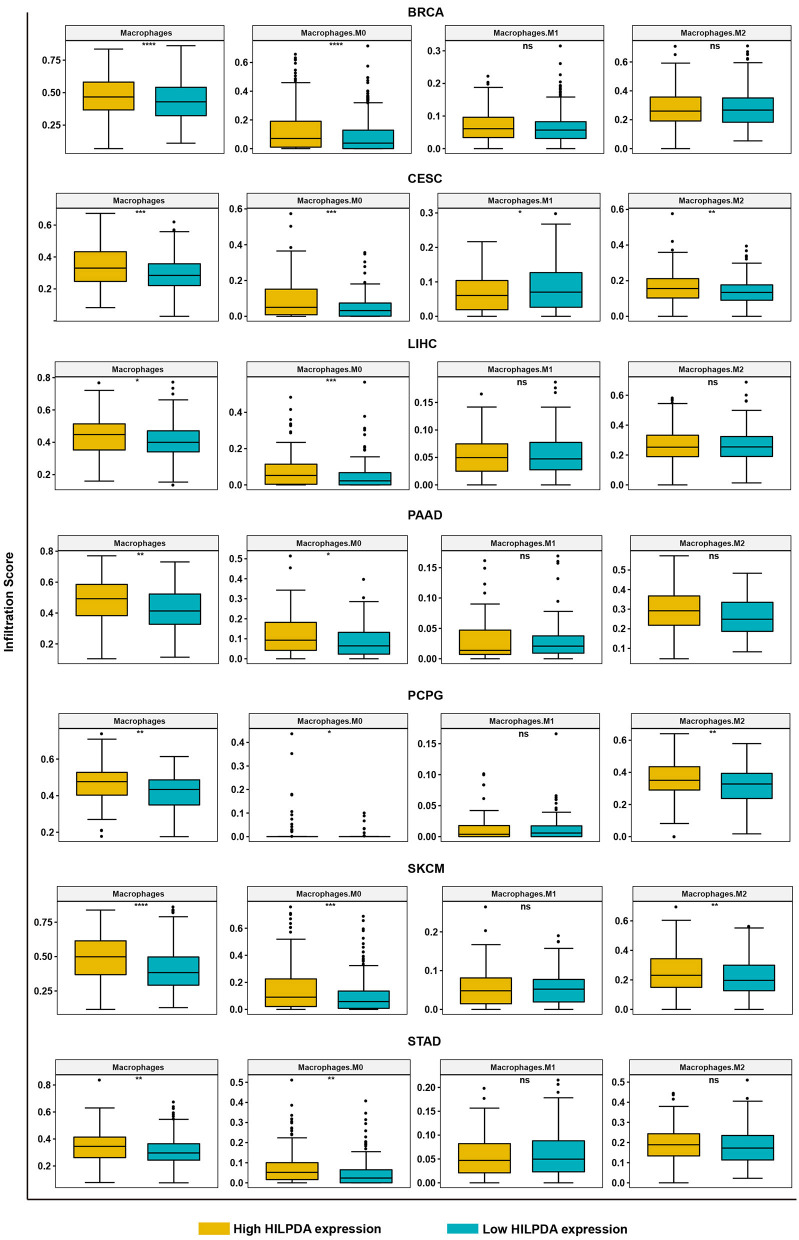
Analysis of TAM infiltration in high and low HILPDA expression groups. The TAM infiltration levels in high HILPDA expression group and low HILPDA expression group in TCGA cohort. Data shown as mean ± SD. *p < 0.05, **p < 0.01, ***p < 0.001, ****p < 0.0001.

**Figure 7:**
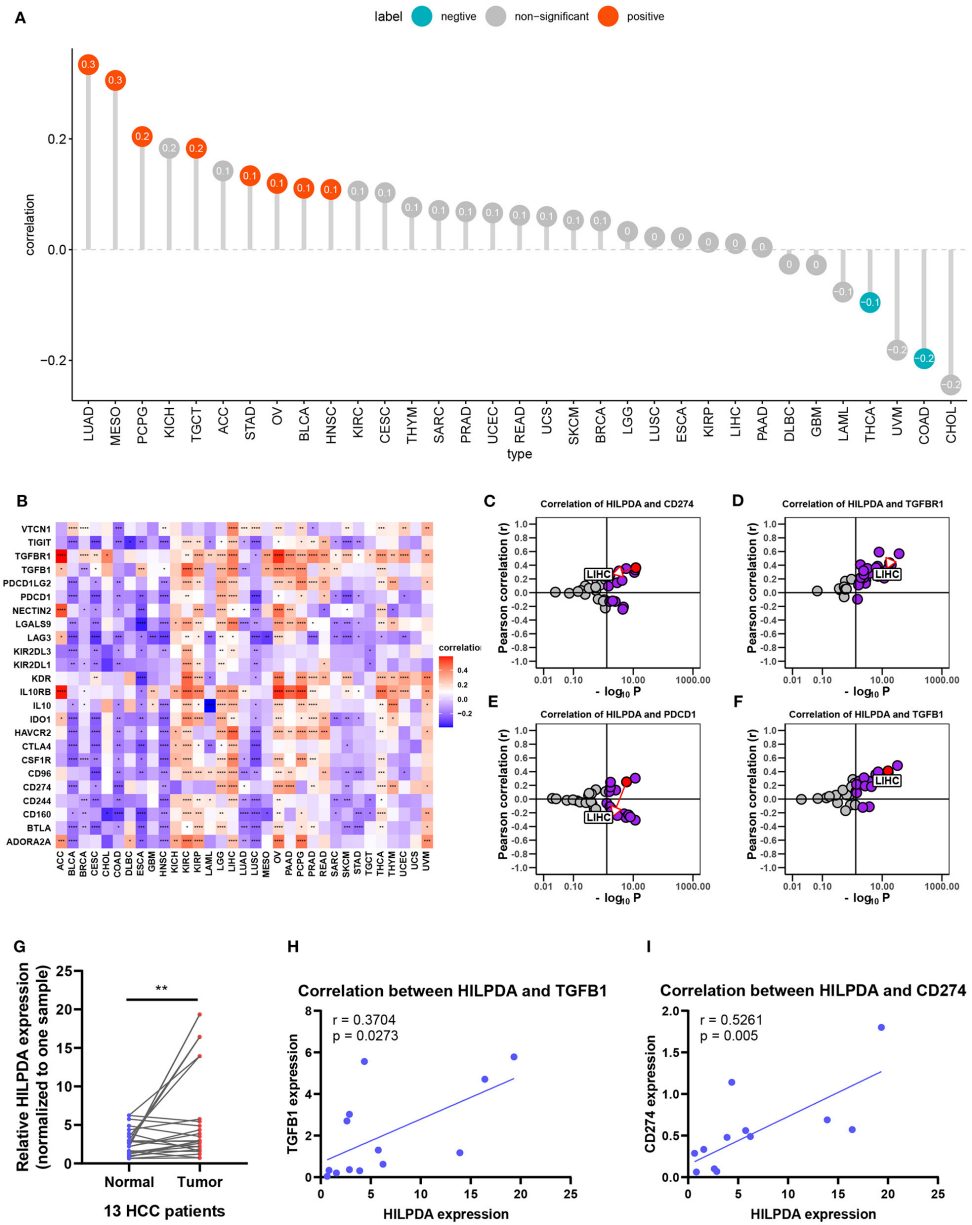
Effects of HILPDA on the immunosuppressive status. **(A)** Correlation between HILPDA and TMB values in LIHC. Red circles represent positive correlation, cyan circles represent negative correlation, and gray circles mean no correlation. The number in the circle represents the correlation coefficient. **(B)** Correlation between HILPDA and immunosuppressive genes is shown in the heatmap, red represents positive correlation, blue represents negative correlation, and the deeper the color, the stronger the correlation. **(C–F)** Correlation coefficient and –log10 (*p*-value) of HILPDA with CD274, TGFBR1, PDCD1, and TGFB1 are shown. Each circle represents a different tumor from TCGA. Red circle is marked for LIHC. Gray circles mean no correlation. **(G)** qRT-PCR results showed the expression of HILPDA in liver cancer tissues. **(H,I)** The correlation between the expression of HILPDA and TGFB1/CD274 based on qRT-PCR results. Data shown as mean ± SD. *p < 0.05, **p < 0.01, ***p < 0.001, ****p < 0.0001.

We further analyzed the effect of HILPDA on the tumor immune microenvironment. We noticed that macrophages, M0 macrophage or M2 macrophage infiltration levels were higher in the high HILPDA expression group in most tumor types, especially in BRCA, CESC, LIHC, PAAD, PCPG, SKCM, and STAD (Figure 6). Correlation analysis revealed that HILPDA expression was positively correlated with infiltration levels of macrophages in most tumor types, including BRCA, CESC, LIHC, PAAD, PCPG, SKCM, STAD, and UCS (Supplementary Figure 1). Tumor-associated macrophage (TAM) infiltration and TMB status are closely related to the immunosuppressive state of the tumor (8). We further analyzed the relationship of HILPDA with TMB and immunosuppressive genes using TCGA pan-cancer data. As shown in Figure 7A, HILPDA expression was positively correlated with TMB in LUAD, MESO, PCPG, TGCT, STAD, OV, BLCA, and HNSC, and negatively correlated with TMB in THCA and COAD. In addition, HILPDA expression was positively correlated with immunosuppressive genes, especially *PD-L1* (*CD274*), *PD-1* (*PDCD1*), *TGFB1*, and *TGFBR1*, in most tumors (Figures 7B–F). Moreover, we validated the expression of HILPDA and the correlation between the expression of HILPDA and TGFB1/CD274 using 13 paired samples from liver cancer patients. The results revealed that HILPDA was highly expressed in liver cancer tissues (Figure 7G). HILPDA expression was positively correlated with TGFB1 and CD274 expression in liver cancer tissues (Figures 7H,I). These results suggest that the high expression of HILPDA is closely related to the immunosuppressive status.

## Discussion

HILPDA is involved in the progression of many diseases, including several tumor types (9, 10). Previous studies have shown the oncogenic role of HILPDA in head and neck carcinoma (10), neuroblastoma (11), and mantle cell lymphoma (12) amongst others. However, HILPDA has not been extensively studied. Therefore, it is urgent to clarify the role of HILPDA in tumor progress and treatment.

In our study, we examined HILPDA expression levels and prognostic function in pan-cancer data using TCGA data from UCSC Xena. Based on our results, we found that HILPDA, compared to normal tissues, was overexpressed in BLCA, BRCA, CHOL, COAD, ESCA, HNSC, KIRC, KIRP, LIHC, LUAD, LUSC, PRAD, READ, and UCEC, while lower expression was observed in THCA in TCGA. The difference in HILPDA expression levels in different tumor types may reflect the distinct underlying functions and mechanisms. We further found that overexpression of HILPDA generally predicts poor prognosis in tumors with high HILPDA expression, such as ACC, KICH, LGG, LIHC, LUAD, MESO, PAAD, and PRAD. These results indicate that HILPDA is a prognostic biomarker for tumor patients.

The tumor microenvironment, especially the immune microenvironment, constitutes a vital element of tumor biology. Increasing evidence has revealed its clinicopathological significance in predicting outcomes and therapeutic efficacy (13, 14). The infiltration of TAMs facilitates the progression of tumors (15, 16). Our results proved that HILPDA has a close relationship with TAM infiltration as TAM infiltration levels were significantly higher in the high HILPDA expression group in BRCA, CESC, LIHC, PAAD, PCPG, SKCM, and STAD. Moreover, HILPDA expression was positively correlated with TAM infiltration level in most tumor types. As the high infiltration of TAMs in tumor often indicates the immunosuppressive microenvironment (17), we further investigated the relationship between HILPDA expression and tumor immunosuppressive microenvironment. We found the positive correlation between HILPDA expression and immunosuppressive genes, such as *PD-L1, PD-1, TGFB1*, and *TGFBR1*, indicates the key role of HILPDA in regulating tumor immunosuppressive microenvironment. The high expression of HILPDA indicates the immunosuppression of most tumors, providing a potential target for immunotherapy.

In summary, we demonstrate that TAM infiltration was significantly increased in tissues with high HILPDA expression and that HILPDA positively correlated with immunosuppressive genes. Our results offer novel insights into the functional role of HILPDA and further highlight a potential mechanistic basis whereby HILPDA influences TAM infiltration and immunosuppressive gene expression in the tumor microenvironment. Collectively, our findings show that HILPDA could be a valuable prognostic biomarker and a potential target for immunotherapy.

## Data Availability Statement

The original contributions presented in the study are included in the article/Supplementary Material, further inquiries can be directed to the corresponding author/s.

## Ethics Statement

The studies involving human participants were reviewed and approved by The Institutional Review Board of Nanfang Hospital, Southern Medical University of Guangdong, China. The patients/participants provided their written informed consent to participate in this study.

## Author Contributions

LL, DW, CL, and XZ designed the study. CL and XZ performed the data analysis. CL and HZ wrote the manuscript and helped with the validation. All authors contributed to the article and approved the submitted version.

## Conflict of Interest

The authors declare that the research was conducted in the absence of any commercial or financial relationships that could be construed as a potential conflict of interest.

## Supplementary Material

Supplementary Figure 1 Correlation analysis between TAM infiltration and HILPDA expression. Plots represent correlation analyses between HILPDA and TAM infiltration in indicated tumor types.

Supplementary Table 1 Pancancer TMB results.

## Abbreviations

ACC: Adrenocortical carcinoma
BLCA: Bladder Urothelial Carcinoma
BRCA: Breast invasive carcinoma
CESC: Cervical squamous cell carcinoma and endocervical adenocarcinoma
CHOL: Cholangiocarcinoma
COAD: Colon adenocarcinoma
DLBC: Lymphoid Neoplasm Diffuse Large B-cell Lymphoma
ESCA: Esophageal carcinoma
GBM: Glioblastoma multiforme
HNSC: Head and Neck squamous cell carcinoma
KICH: Kidney Chromophobe
KIRC: Kidney renal clear cell carcinoma
KIRP: Kidney renal papillary cell carcinoma
LAML: Acute Myeloid Leukemia
LGG: Lower Grade GLioma
LIHC: Liver hepatocellular carcinoma
LUAD: Lung adenocarcinoma
LUSC: Lung squamous cell carcinoma
MESO: Mesothelioma
OV: Ovarian serous cystadenocarcinoma
PAAD: Pancreatic adenocarcinoma
PCPG: Pheochromocytoma and Paraganglioma
PRAD: Prostate adenocarcinoma
READ: Rectum adenocarcinoma
SARC: Sarcoma
SKCM: Skin Cutaneous Melanoma
STAD: Stomach adenocarcinoma
TGCT: Testicular Germ Cell Tumor
THCA: Thyroid carcinoma
THYM: Thymoma
UCEC: Uterine Corpus Endometrial Carcinoma
UCS: Uterine Carcinosarcoma
UVM: Uveal Melanoma.
