# Supplementary figures and images for: HILPDA Is a Prognostic Biomarker and Correlates With Macrophage Infiltration in Pan-Cancer
Source: Front Oncol. 2021 Mar 18;11:597860. doi: 10.3389/fonc.2021.597860 (PMC8015804; doi:10.3389/fonc.2021.597860)

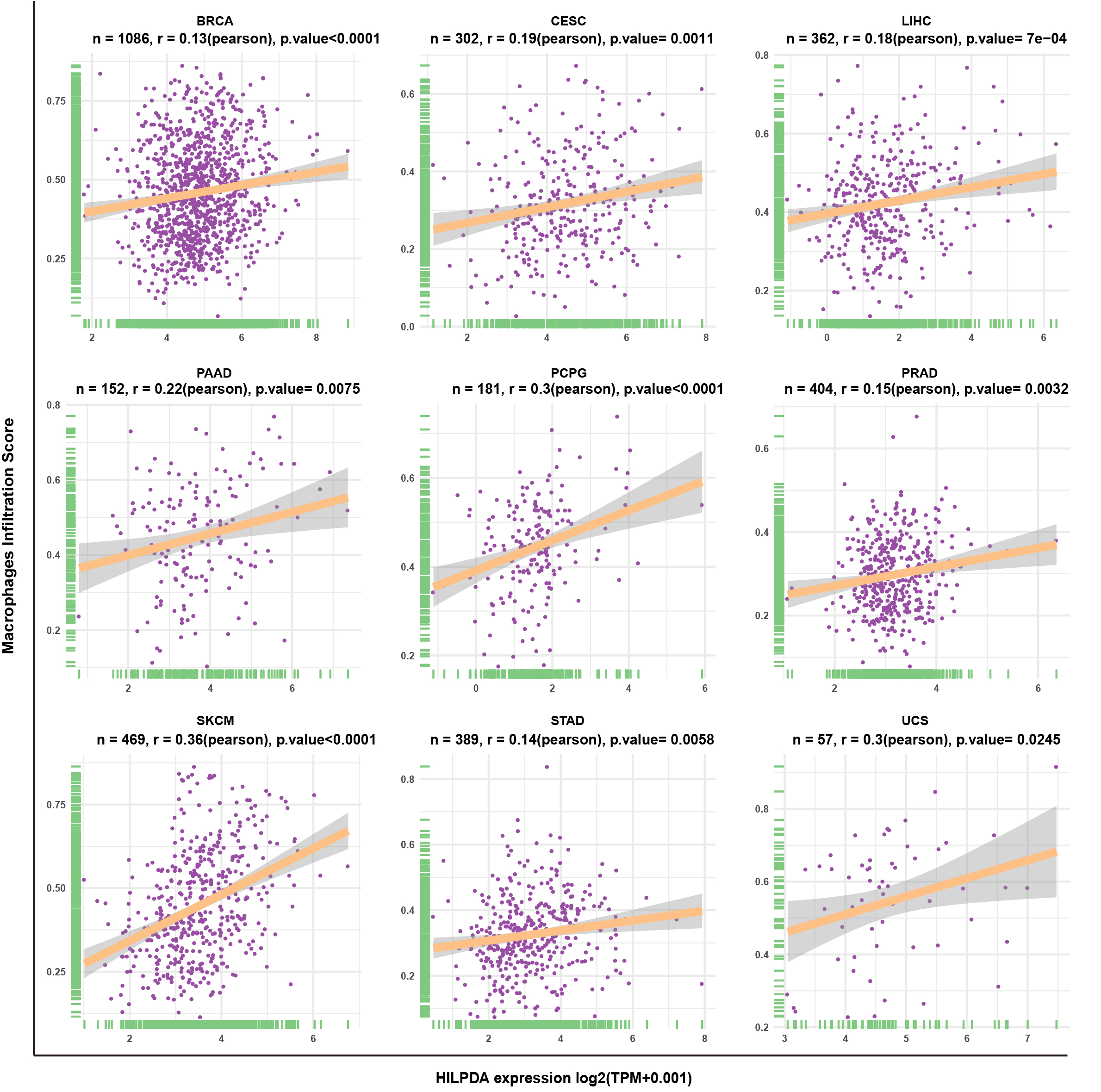

Supplement: Supplementary Figure 1 — Correlation analysis between TAM infiltration and HILPDA expression. Plots represent correlation analyses between HILPDA and TAM infiltration in indicated tumor types. [file Image_1.jpg]
